# A step‐by‐step protocol for meiotic chromosome counts in flowering plants: A powerful and economical technique revisited

**DOI:** 10.1002/aps3.11342

**Published:** 2020-04-23

**Authors:** Michael D. Windham, Kathleen M. Pryer, Derick B. Poindexter, Fay‐Wei Li, Carl J. Rothfels, James B. Beck

**Affiliations:** ^1^ Department of Biology Duke University Campus Box 90338 Durham North Carolina 27708 USA; ^2^ Department of Biology University of North Carolina at Chapel Hill Coker Hall, 120 South Road Chapel Hill North Carolina 27599 USA; ^3^ Boyce Thompson Institute and Cornell University 533 Tower Road Ithaca New York 14853 USA; ^4^ University Herbarium and Department of Integrative Biology University of California Berkeley 3040 Valley Life Sciences Building Berkeley California 94720 USA; ^5^ Department of Biological Sciences Wichita State University 537 Hubbard Hall Wichita Kansas 67260 USA; ^6^ Botanical Research Institute of Texas 1700 University Drive Fort Worth Texas 76107 USA

**Keywords:** chromosome number, chromosome squash, cytology, meiosis, polyploidy, species complex

## Abstract

**Premise:**

Counting chromosomes is a fundamental botanical technique, yet it is often intimidating and increasingly sidestepped. Once mastered, the basic protocol can be applied to a broad range of taxa and research questions. It also reveals an aspect of the plant genome that is accessible with only the most basic of resources—access to a microscope with 1000× magnification is the most limiting factor.

**Methods and Results:**

Here we provide a detailed protocol for choosing, staining, and squashing angiosperm pollen mother cells. The protocol is supplemented by figures and two demonstration videos.

**Conclusions:**

The protocol we provide will hopefully demystify and reinvigorate a powerful and once commonplace botanical technique that is available to researchers regardless of their location and resources.

Plant chromosome number is highly variable, ranging from as few as four chromosomes per somatic cell (*Xanthisma gracile* (Nutt.) D. R. Morgan & R. L. Hartm., Asteraceae) to as many as 1440 (*Ophioglossum reticulatum* L., Ophioglossaceae). Changes in chromosome number and the genomic rearrangements that usually accompany this transition (Madlung and Wendel, 2013) have profound effects on plant phenotypes (Levin, 2002; Otto, 2007; Parisod, 2012). Such changes can result in rapid reproductive isolation, aiding in the establishment of lineages with novel genomes and phenotypes (Ramsey and Schemske, 2002). Indeed, whole‐genome duplication (polyploidization) is associated with approximately 15% of angiosperm speciation events and 30% of fern speciation events (Wood et al., 2009). At the macro‐evolutionary level, polyploidization episodes have been inferred throughout much of the evolution of plants (Leebens‐Mack et al., 2019).

A traditional way to observe chromosome number (a “chromosome count”), morphology, and pairing behavior is through a chromosome “squash.” Cells undergoing meiosis or mitosis are isolated, stained, and flattened, allowing condensed chromosomes to be observed and counted. Although chromosome counts continue to be reported (Cheema et al., 2019; Sadeghian et al., 2019; Schilling et al., 2019), it has been suggested that fewer young researchers are being trained in these methods (Goldblatt, 2007). This has contributed to a recent decline in published chromosome counts and an even sharper reduction in the number of publications per year providing these new data. The overall pattern is well illustrated by counts for *Draba* L., the largest genus of the Brassicaceae (Fig. 1A). In this predominantly temperate/boreal group, the number of published counts per year peaked in 1966 (36 taxa in six publications), while the number of publications containing new *Draba* counts peaked in the early 1970s (nine publications each in 1972 and 1974). The exact timing of these research peaks differs from one taxonomic group to another, with tropical groups often lagging behind temperate taxa. For example, in the genus *Dendrobium* Sw. (one of the largest genera of Orchidaceae), the number of published counts per year peaked in 1982 (113 taxa in three publications), while the number of publications containing new *Dendrobium* counts peaked slightly earlier (four publications each in 1970, 1976, and 1980; Fig. 1B). Both genera exhibit relatively little activity in the past decade despite the fact that fewer than half the described species have been counted.

**Figure 1:**
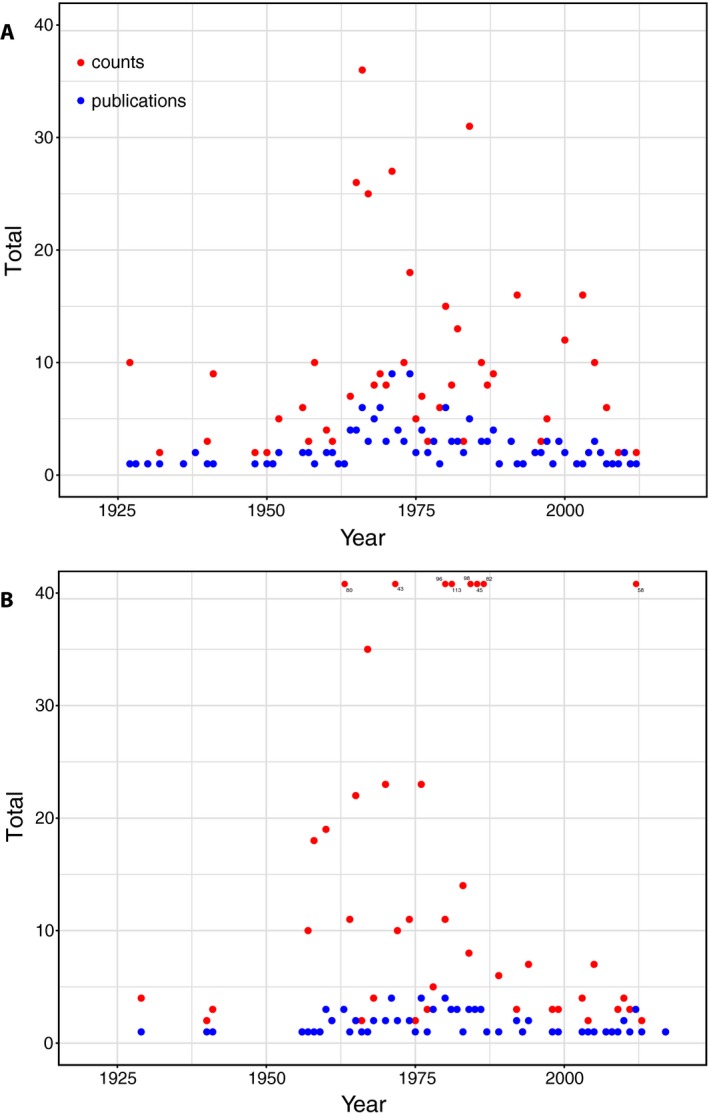
Chromosome count reporting through time in two large angiosperm genera. (A) The number of unique publications reporting new chromosome counts from *Draba* by year (1920–2019), and the total number of counts reported by these publications by year. (B) The number of unique publications reporting new chromosome counts from *Dendrobium* by year (1920–2019), and the total number of counts reported by these publications by year. Note that the number of *Dendrobium* counts was >40 in eight years, and in these cases the number of counts is noted with a sub‐ or superscript. Counts published during the years 1920–1965 were obtained from *Chromosome Numbers of Flowering Plants* (Federov, 1974), and those from 1966–2003 were assembled from the series *Index to Plant Chromosome Numbers* (Goldblatt, 2007 and references cited therein). The Chromosome Counts Database (Rice et al., 2015; http://ccdb.tau.ac.il) and Google Scholar were searched to identify counts published from 2004–2019.

One factor contributing to the decline of chromosome counting is the increasingly widespread use of flow cytometry to estimate ploidy, the number of chromosome sets present in an individual. Flow cytometry allows the genomic DNA content of nuclei to be estimated (Kron et al., 2007). Ploidy can be inferred from these data, but all such inferences generated using flow cytometry are relative unless calibrated by chromosome counts derived from the same individual through a traditional chromosome squash (Suda et al., 2007). Given the documented variability of genomic DNA content within genera (Leitch et al., 2019), uncalibrated flow cytometry inferences of ploidy should be (cautiously) used only when comparing close relatives or tissues from a single individual. Some genera (most notably *Carex* L.) exhibit extensive intraspecific chromosome number variation without appreciable changes in total genome size (Chung et al., 2011; Escudero et al., 2015). This stands in sharp contrast to the situation in *Equisetum* L. (Equisetaceae), in which genomic DNA content differs more than two‐fold between its two largest subgenera, *Equisetum* and *Hippochaete* (Obermayer et al., 2002). This difference would be misconstrued as ploidy variation if one were to base their conclusions on flow cytometry alone, and it is only through the application of traditional cytogenetic methods that we know these sister lineages have the same chromosome number (2*n* = 216), but distinctly different chromosome sizes (Manton, 1950).

In addition to providing powerful data themselves and allowing for flow cytometry calibration, traditional chromosome squashes are also economical. The purchase price of a flow cytometer (ca. US$50,000) is not feasible for many researchers, not to mention the related expenses throughout the lifetime of the device (e.g., supplies, reagents, maintenance, repairs). A quality phase‐contrast microscope equipped with a digital camera is ca. US$6000, with only modest lifetime costs for maintenance and supplies. Critically, many biology departments already possess appropriate microscopes and supplies for existing teaching and research purposes, rendering the techniques described herein nearly cost‐free.

The perceived difficulty of chromosome counts via the squash technique has also contributed to the decline in its use (Windham and Yatskievych, 2003; Goldblatt, 2007). This barrier is exacerbated by a lack of sufficient detail on critical aspects of the protocol (e.g., tissue choice, maceration, squashing) in most cytogenetic publications. Here, we provide a detailed guide to choosing, staining, and squashing angiosperm pollen mother cells. The target are cells undergoing meiosis; hence, this is a “meiotic count” protocol. Squashes targeting cells undergoing mitosis can also be performed, and an explicit published protocol for this method is also badly needed. The meiotic count protocol we present is a modified version of the acetocarmine squash technique (Belling, 1921; Smith, 1947), a classic method that has been applied to a wide range of plant taxa (Sharma and Sharma, 1965), including ferns (Manton, 1950; Britton, 1953).

## METHODS AND RESULTS

The staining and squashing portion of the protocol (steps 5–20) usually requires 1–2 hours, depending on familiarity with both the technique and the taxon under study. Although one or more unsuccessful (i.e., uncountable) preparations are often needed to identify appropriate bud sizes (step 7), subsequent preparations of the same taxon are generally successful. The steps below are demonstrated in Videos S1 and S2, with relevant portions noted below. A list of required materials and reagents is provided in Appendix 1. A list of commonly encountered issues and potential solutions is provided in Appendix 2.

### Sampling tissues at the right developmental stages (Video S1, 1:20–2:35)

#### Step 1

It is important to sample across the full range of flower bud ages, erring toward younger material. Most older buds are long past active meiosis, and failing to sample sufficiently young material is one of the most common mistakes made. Whenever possible, sample whole or partial inflorescences; knowledge of the arrangement of buds relative to one another will greatly simplify later efforts to locate anthers at the proper stage of meiosis. In some taxa, you will be able to sample a large amount of appropriate material from the same individual. These taxa include species that feature large and/or many inflorescences presenting a range of bud sizes at any given time. In many cases, however, material from multiple individuals will need to be sampled in order to ensure that a count can be obtained. In these cases, it is important to sample from as small a number of closely neighboring individuals as possible. If one does sample from multiple individuals, any variation is likely to be apparent on the slide, as several buds are disrupted, stained, and examined simultaneously in each squash.

### Fixing material (Video S1, 2:35–7:00)

#### Step 2

In the first step of the fixing process, tissues should be placed in a 3 : 1 95% ethanol : glacial acetic acid solution, known as Farmer's fixative, which should be freshly prepared on the day of use and preferably kept on ice before and after use. The substitution of this solution for Carnoy's fixative (6 : 3 : 1 95% ethanol : chloroform : glacial acetic acid) is recommended for plants in which the anthers are enclosed by tissues that are physically or chemically resistant to fixative penetration. Any vial with a secure gasket seal should suffice, and 20‐mL Wheaton scintillation vials with cone cap liners work well. For best results, the fixed buds should occupy no more than 50% of a full vial of fixative.

#### Step 3

A voucher specimen should be prepared from the sampled individual(s) and the collector/collection number recorded both on the lid of the vial and, in pencil, on a small paper slip that is kept inside the vial.

#### Step 4

After waiting 24 hours, the fixative can be poured off, replaced with 70% ethanol, and placed in a −20° freezer. Materials fixed and stored in this manner can last many years.

### Choosing material to squash (Video S2, 2:55–7:40)

#### Step 5

To prepare the material for the squash, first use Kimwipes and ethanol to thoroughly clean a standard 75 × 25‐mm microscope slide and 30 × 22‐mm cover slip. Note that these cover slips are larger than the more common 22 × 22‐mm size. All fingerprints, lint, and dust particles need to be removed to facilitate good squashes. Label the slide with the date and collection number to maintain a link to the voucher specimen.

#### Step 6

Forceps can then be used to transfer the fixed materials from their storage in 70% ethanol to a clean glass Petri dish. An array of bud sizes should be transferred and submerged in a small amount of 70% ethanol to keep the sample from drying out.

#### Step 7

Under a dissecting microscope, buds of appropriate size should be selected for anther extraction. When initiating work on a new species, it is best to start with the youngest flower buds available. First open a bud and remove an anther; if the latter shows any coloration other than white, it is likely too old. If the excised anther is white and normally developed, gently break it open with a needle tip under high magnification. If the contents consist of abundant, isolated, glassy‐looking cells, the bud is post‐meiotic. Next, move to a slightly smaller bud and repeat until the anther contents emerge as irregular clumps of cells. If any tissues can be easily separated from the anthers, do this now in ethanol in the Petri dish with sharpened dissecting needles and/or razor blades. The anthers are the sole target of this effort; the more that can be isolated from other floral structures, the better.

### Isolating material (Video S2, 7:40–14:00)

#### Step 8

**Figure 2:**
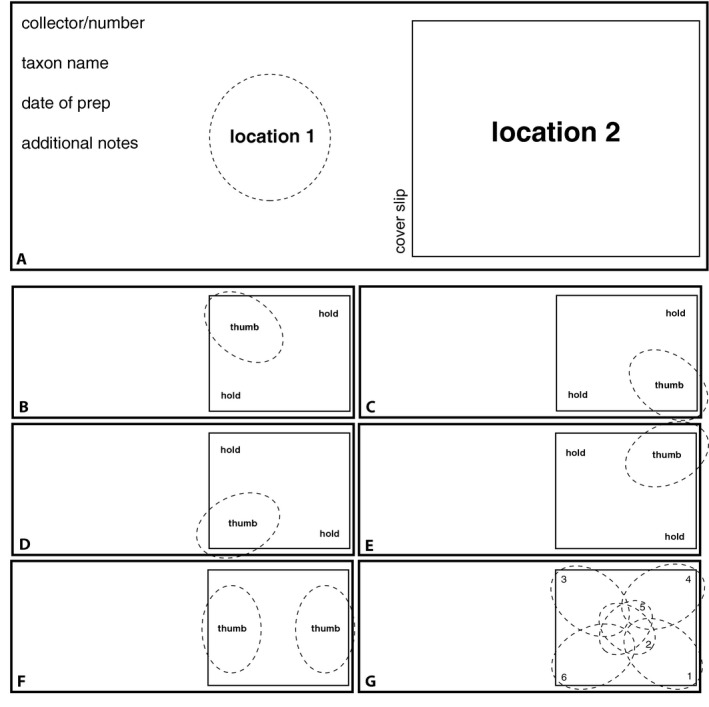
The slide working area and strategies for squashing. (A) Layout of a typical slide, showing the location of the dilute acetocarmine stain (location 1) and the full‐strength acetocarmine stain (location 2). (B–F) The position of the non‐dominant thumb/forefinger (“hold”) and dominant thumb during the five squashing steps described in the manuscript. (G) An alternative succession of thumb positions.

A clean microscope slide should be placed on a light‐colored background under a dissecting microscope. If you are right‐handed, position a droplet of dilute acetocarmine stain (for recipe, see Video S1: 9:10–10:10) on the left side of the clean slide (location 1 in Fig. 2A). Do this by closing the forceps, dipping them into the dilute stain bottle, then touching the slide at location 1 and releasing the forceps tips. Transfer the materials isolated in step 7 into this droplet.

#### Step 9

Under the dissecting microscope, further isolate the anthers from the transferred material. Complete isolation will be possible for some taxa, but for others, only the removal of certain accessory structures (i.e., pappus and immature cypselae in Asteraceae) may be feasible given time constraints. As non‐anther material is removed, use your dissecting needles to push these tissues aside and gather the anthers into a small pile in the center of the dilute acetocarmine stain droplet. The forceps can be used to add small amounts of dilute acetocarmine to the edge of the droplet to counter shrinkage due to evaporation; no area containing anthers intended for squashing should be allowed to dry out.

#### Step 10

The optimal number of anthers per slide varies widely based on the size of the floral structures involved, but the amount of plant material in the droplet should not exceed 10% of its volume. Anthers of slightly different sizes and/or derived from more than one bud should be combined in one droplet to improve the likelihood of encountering cells at the correct stage of meiosis. Once the anther sample has been assembled, place a small droplet of full‐strength acetocarmine (for recipe, see Video S1: 9:10–10:10) near the right end of the slide (location 2 in Fig. 2A). As before, do this by dipping the forceps into the stain bottle and then touching the slide at location 2 with the tips of the forceps. Apply as small a droplet of full‐strength acetocarmine as is feasible; try to create a shallow pool <5 mm in diameter. The dissected anthers can then be transferred from location 1 to this droplet and mixed into the stain. The droplet of dilute acetocarmine at location 1 will be relatively small at this point due to evaporation, and all anthers can be moved to the stain droplet at location 2 using the tip of a dissecting needle. Note that once you begin working in the full‐strength acetocarmine, the preparation becomes more time sensitive due to the rapid evaporation of the stain. Thus, it is important that all downstream reagents and materials are ready before moving to full‐strength stain.

### Disrupting tissue and removing large pieces (Video S2, 14:00–27:25)

#### Step 11

With the sample suspended in the thin film of full‐strength acetocarmine, a dissecting needle should be held nearly at horizontal and used to crush the anthers. Crushing when the droplet is relatively small is important, as this will minimize how far the acetocarmine droplet spreads beyond its initial boundaries. The stain should not dry out at any point during this process. When necessary, a small amount of additional full‐strength acetocarmine can be added with forceps to moisten the drying edges. Precipitated stain around the edges of the droplet should be resuspended by stirring while simultaneously keeping the droplet as small as possible. Repeat this process (add stain, crush anthers, stir, etc.) until the majority of the sample cannot be homogenized further.

#### Step 12

At this point, any remaining unhomogenized material can be gathered into a pile at the edge of droplet and removed with forceps or dissecting needles. If left in the droplet, these larger pieces will interfere with your ability to fully flatten the cells. As before, add stain as needed to prevent any area occupied by the droplet from drying out. The final droplet size should be no more than 1 cm in diameter.

#### Step 13

Using a dissecting needle, add a droplet of Hoyer's solution (Hempstead Halide, Galveston, Texas, USA) about equal in volume to the stain droplet and mix the acetocarmine, Hoyer's, and stained material thoroughly. Hoyer's solution (Anderson, 1954) is a mounting and preserving medium that reduces cover slip rebound after squashing and improves chromosome visibility by partially de‐staining the cell cytoplasm. Note that although gloves were not worn in the accompanying video, they are recommended during this and subsequent steps due to the toxicity of Hoyer's solution.

### Adding the cover slip and squashing (Video S2, 27:25–33:30)

#### Step 14

With the microscope slide sitting on a light‐colored background under a dissecting microscope, the cleaned cover slip should be centered over the acetocarmine/Hoyer's mixture and lowered into position. If the droplet spreads slowly to the edges of the cover slip, the mixture of Hoyer's solution and stain is about right. If it races to the edges, the amount of acetocarmine relative to Hoyer's solution will need to be reduced in future preparations. If it fails to reach the edges of the cover slip, less Hoyer's solution should be added in future preparations.

#### Step 15

While securing the cover slip in place with a gloved fingertip, the cover slip should be tapped gently with a dissecting needle to disperse cells and release bubbles from under the cover slip. Any bubbles visible at 10× magnification should be removed if possible, although this may be difficult if unhomogenized tissues are present.

#### Step 16

Pick a flat surface just below your waist height and ensure that it is free of any dirt or sand. The slide should be placed in the fold of a paper towel near the edge of this surface. Gently press the cover slip to push excess liquid to the edges. This excess will seep through the towel and reveal the edges of the cover slip to facilitate proper positioning of your fingers and thumbs in steps 17 and 18.

#### Step 17

Place the thumb and index finger of your non‐dominant hand on two opposite corners of the cover slip and press straight down on one of the two open corners with the thumb of your dominant hand (Fig. 2B). Press *straight down* with as much pressure as you can while using the thumb and index finger of your non‐dominant hand to prevent the cover slip from moving. Strictly vertical pressure with minimal side slippage is critical. Press for 15 seconds, gently release, then press on the other open corner for 15 seconds (Fig. 2C). Switch your non‐dominant thumb and index finger to the two corners you just pressed and press for 15 seconds each on the two open corners (Fig. 2D, E).

#### Step 18

Next, turn the slide so you can comfortably place both thumbs on the short edges of the cover slip (Fig. 2F). Simultaneously (both thumbs at once) press for 15 seconds on these portions. This step is designed to additionally flatten the short edges of the preparation, which is where most countable cells will be found. Note that numerous strategies for thumb placement and order exist, such as the alternative presented in Fig. 2G. You will optimize what works best for you through trial and error.

### Slide cleanup and sealing (Video S2, 33:30–37:10)

#### Step 19

Remove the slide from the folded paper towel and place it over a clean section of the towel. Bubbles may appear under the cover slip, with more being drawn in along the edges by cover slip rebound. To counter this, the tip of a dissecting needle should be dipped in Hoyer's solution and used to transfer a small droplet to the edge of the slip where bubbles are forming, dragging the excess along that edge. Repeat for all edges, wiping the dissecting needle between dips. This small bead of Hoyer's solution along the cover slip edge secures the slip and prevents additional air from entering. Use as little as possible, as excess Hoyer's will seep under the slip and overly lighten the stained cells in that critical edge region.

#### Step 20

Finally, a Kimwipe or paper towel should be folded into a small point, moistened with ethanol, and used to wipe any excess Hoyer's solution away from the surface of the cover slip. A single motion should be used to swipe toward the edge of the slip, and the Kimwipe should be changed after each swipe. Clean until no streaks are visible, as Hoyer's on the cover slip surface will hinder examination of that area.

### Slide scanning and storage

To identify countable chromosomes, carefully scan the slide using a medium power (40×) objective, preferably on a phase‐contrast microscope equipped with a digital camera. Start at the top corner of one of the short edges of the cover slip. Scan down until you encounter the bottom edge. Move the field of view to the side a bit and scan upwards. Repeat this up and down process, moving across the slide. Note that while you can scan left to right, we have found scanning up and down reduces nausea. Identify cells that are worth revisiting later at higher magnification by recording the X/Y coordinates on the microscope stage. Note that most countable cells will be near the edges of the cover slip due to a greater concentration of cells near the cover slip center. The slip will be less flattened near the center, and it will be difficult to view all chromosomes of a single cell simultaneously in one focal plane. If an abundance of well‐squashed cells are found in the first few scans, you can switch to the 100× objective; however, we generally recommend scanning the entire slide. The 100× objective requires applying immersion oil to the cover slip, which limits the future use of lower power objectives unless the slide is removed and cleaned. Take images of a variety of cells exhibiting countable chromosomes, saving each image using the sample's collector/collection number and X/Y coordinates of the cell as the image name. When this process is complete, carefully remove the immersion oil from the cover slip with an ethanol‐moistened Kimwipe and store in a slide storage cabinet, preferably horizontally. Slides prepared and stored in this way will remain viewable for several months, perhaps longer.

### Basic data interpretation

Any anther can yield at least a few cells with condensed, stainable chromosomes. Very young anthers will show only mitotic cell divisions (often in abundance); the same is true of older anthers in which mitotic cell divisions are concentrated in the tapetum and maturing pollen grains. At the peak of microsporogenesis, cells undergoing meiotic divisions far outnumber those dividing by mitosis, but the latter are always present to some degree. Therefore, the first step in interpreting plant chromosome squashes involves being able to distinguish between cells undergoing meiotic and mitotic cell divisions.

**Figure 3:**
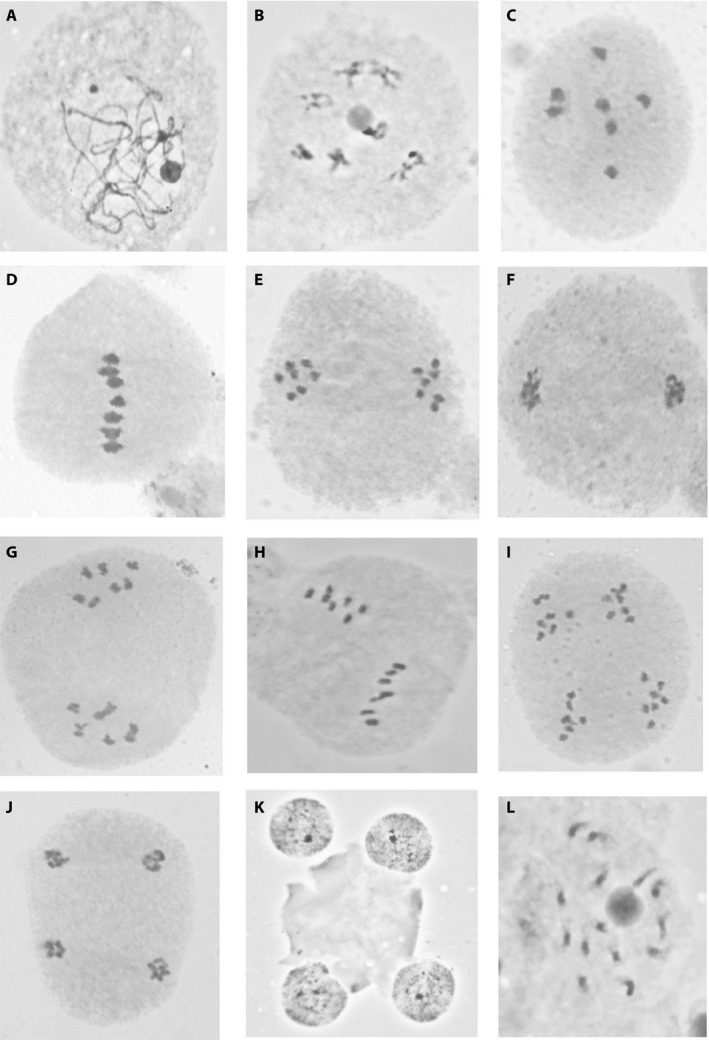
Stages of meiosis (A–K) and mitosis (L), as documented with acetocarmine squashes of *Boechera* (Brassicaceae). (A) Early prophase I. (B) Mid prophase I. (C) Diakinesis (very late prophase I). (D) Metaphase I. (E) Late anaphase I. (F) Telophase I. (G) Prophase II. (H) Metaphase II. (I) Anaphase II. (J) Telophase II. (K) Microspore tetrad. (L) Mitotic late prophase. The largest chromosome in panel D is ca. 1 μm in length.

Figure 3 provides a visual guide to aid in distinguishing meiosis (Fig. 3A–K) from mitosis (Fig. 3L) and categorizing the various stages of the former. The photos all derive from sexual diploid (*n* = 7, 2*n* = 14) species of *Boechera* Á. Löve & D. Löve (Brassicaceae), but the critical distinguishing features apply to the majority of land plants. When discriminating between meiosis and mitosis, note that mitotic chromosomes never condense as fully as meiotic chromosomes. Even as they approach the point of maximum condensation, mitotic chromosomes remain more elongate and amorphous, with the chromosome arms appearing as faint, fuzzy terminal regions (Fig. 3L). By contrast, the same set of 14 unpaired chromosomes at the analogous stage (late prophase) of meiosis II are much more condensed and sharply defined (Fig. 3G). The presence of a nucleolus in mitotic cells (circular stained area among chromosomes in Fig. 3L) also serves to differentiate them from many cells undergoing meiotic division. The only stage of meiosis in which evident nucleoli co‐occur with discrete, countable chromosomes is mid‐ to late prophase I (Fig. 3B), and the classic “X‐ and O‐shaped” paired chromosomes of these cells are easily distinguished from the small, amorphous, unpaired chromosomes characteristic of mitosis (Fig. 3L). Note, however, that the transition from these X‐ and O‐shaped chromosomes (Fig. 3B) to more condensed ones typical of diakinesis (very late prophase I; Fig. 3C) is gradual, and cells with fairly condensed chromosomes, even similar to those in Fig. 3C, can be observed at times.

Meiotic chromosome squash cells should ideally be observed at a variety of stages, and it is critical to know exactly which phases are represented on your slide. The most easily interpreted stage of meiosis is typically diakinesis (very late prophase I; Fig. 3C). Here the chromosomes are tightly paired (so the number of observed units is at its lowest; the *n* number), maximally condensed, and scattered throughout the cytoplasm (so there is less potential for overlap), and the only other dark staining body in the cell that might be mistaken for a chromosome (the nucleolus present at earlier stages) has dissipated. Metaphase I (Fig. 3D) has many of the same advantages, except that the close proximity of the chromosome pairs aligned along the cell equator means that overlap can be a problem, especially for organisms with higher chromosome numbers. Cells at anaphase I (Fig. 3E), late prophase II (Fig. 3G), and metaphase II (Fig. 3H) are also countable, but it is imperative to recognize that there are two clusters of unpaired chromosomes within the undivided cell for these post–metaphase I squashes to be correctly interpreted (in our example, *n* = 7, 2*n* = 14, rather than *n* = 14). Squashes of cells in anaphase II (Fig. 3I) occasionally contribute useful information, but the presence of four spatially discrete chromatid clusters must be noted to prevent misinterpretation. If cells are squashed in non‐classic orientations (e.g., polar rather than equatorial views), the relative size of the stained bodies can help distinguish among the six stages of meiosis in which DNA is fully condensed. The paired chromosomes of very late prophase I (Fig. 3C) and metaphase I (Fig. 3D) are about twice the size of the unpaired chromosomes of anaphase I (Fig. 3E), late prophase II (Fig. 3G), and metaphase II (Fig. 3H), and the latter are about twice the size of the individual chromatids observed in anaphase II (Fig. 3I). It is very rare to be able to count chromosomes at early prophase I (Fig. 3A), telophase I (Fig. 3F), telophase II (Fig. 3J), or at the microspore tetrad stage (Fig. 3K); however, the presence of any of these stages on your slide is promising, as it indicates that the tissue you prepared is undergoing meiosis.

## CONCLUSIONS

Traditional chromosome squashes are the quickest, least expensive way for a researcher to obtain chromosome counts and observe chromosome morphology/pairing behavior. This technique therefore remains an indispensable part of the analytical toolkit in plant systematics and evolution. We encourage all botanists, particularly those who are early‐career researchers, to apply this and other classic protocols to their study system.

## Supporting information


**VIDEO S1.** Fixing material and reagent preparation. This video is also available at: www.youtube.com/watch?v=iXqni6knH5A.

**VIDEO S2.** Choosing, staining, and squashing material. This video is also available at: www.youtube.com/watch?v=xVV4qBfSQLs.

## APPENDIX 1 Required materials/reagents to perform meiotic chromosome counts in flowering plants. Supplier and part number are noted for specific items.

### Chemicals/solutions

95% ethanol

Glacial acetic acid

Carmine (Fisher Scientific #C579‐25)

Hoyer's solution (Hempstead Halide, Galveston, Texas, USA)

Immersion oil (Fisher Scientific #12‐070‐396)

### Supplies

20‐mL scintillation vials (DWK Scientific, Mainz, Germany, #986736)

Kimwipes (Fisher Scientific, Hampton, New Hamphsire, USA #06‐666A)

75 × 25 mm microscope slides

30 × 22 mm microscope slide cover slips (Fisher Scientific #12‐545‐A)

Forceps

Dissecting needles

Glass Petri dish

### Equipment

−20° freezer

Dissecting microscope

Phase‐contrast microscope with a mounted digital camera

## APPENDIX 2 Commonly encountered issues and potential solutions.

### Cellular components cannot be visualized because cells are not fully flattened

Potential causes: (A) Certain relatively stiff floral parts (e.g., Asteraceae pappus) were not removed during the dilute stain step (step 9) and cannot be disrupted during later stages. (B) Material was not sufficiently removed from the full‐strength acetocarmine field prior to the addition of Hoyer's solution (step 12). (C) Excessive Hoyer's solution was added prior to squashing (step 13), resulting in an overly viscous mixture. (D) Foreign objects, such as fibers from clothing or lab towels, remained in the preparation. (E) The chosen material (steps 1 and 7) is too old, and mature pollen grains with tough exine layers are present.

All of these factors will prevent the cover slip from being fully pressed against the slide. A common sign that this is occurring is the presence of large bubbles following squashing; these result from bulges in the cover slip that allow air to immediately seep in.

### Cells are insufficiently stained

Potential causes: (A) The full‐strength acetocarmine steps (11–12) were performed too quickly, allowing insufficient time for the stain to penetrate the material. (B) Excessive Hoyer's solution was added prior to squashing (step 13) or along the edges of the slip following squashing (step 19), thus over‐clearing the cells. (C) Time and/or heat is required for satisfactory staining. The cells of some taxa will stain slowly, and in these cases, cells will appear significantly better stained following storage at room temperature for 1–2 days following step 20. The staining of some taxa will also benefit from mild heat. Once the acetocarmine/Hoyer's mixture has been prepared (step 13), place the slide on a slide warmer or hot plate on a very low setting for ca. 10 seconds. The mixture will rapidly evaporate, so take care to not over‐heat.

### Cells are overly stained

Potential causes: (A) Cells either remained in full‐strength stain for too long, were examined after a long storage period, or were over‐exposed to heat (see #2, above). (B) Insufficient Hoyer's solution was added (step 13).

### Few cells are visible on the slide

Potential causes: (A) Too little material was initially added to the preparation (step 7). (B) Material was insufficiently disrupted during step 11. In this case, few individual cells were liberated and very few cells will remain in the field when visible material is removed (step 12).

### Essentially all cells in the field appear largely “blank,” with perhaps only a stained nucleolus visible

Potential cause: (A) Chosen material (steps 1 and 7) is too young, and cells are in interphase.
